# Superior Gluteal Artery Pseudoaneurysm Following a Minor Ground-Level Traumatic Fall

**DOI:** 10.7759/cureus.71762

**Published:** 2024-10-18

**Authors:** Marta B Sekh, Kelon Scott, Tugba Akilli, Maria A Guirguis, Lance Jung, Michael R Zemaitis

**Affiliations:** 1 General Surgery, American University of Antigua College of Medicine, Osbourn, ATG; 2 Pharmacology, Massachusetts College of Pharmacy and Health Sciences, Boston, USA; 3 General Surgery, Richmond University Medical Center, New York, USA; 4 Trauma Surgery, Richmond University Medical Center, New York, USA

**Keywords:** arterial pseudoaneurysms, pseudoaneurysms of the superior gluteal artery, traumatic fall, traumatic gluteal artery pseudoaneurysm, traumatic pseudoaneurysm

## Abstract

Pseudoaneurysms of the superior gluteal artery (SGA) are exceedingly rare, especially following minor trauma. This case report presents a 51-year-old male with a history of aortic valve replacement and daily antiplatelet therapy who developed a pseudoaneurysm of the SGA following a ground-level fall. Due to its subclinical presentation, the patient was initially diagnosed with a hematoma of the right gluteus, which failed to resolve and became symptomatic after six months. During an initial attempt to evacuate the presumed hematoma, an intraoperative hemorrhage led to the discovery of the pseudoaneurysm and required prompt embolization. This case report underscores the importance of considering vascular injury, even after a minor trauma, particularly in patients on antiplatelet therapy. In such patients, a higher index of suspicion might be required for early recognition and appropriate interventions to prevent such life-threatening complications.

## Introduction

In true aneurysms, all three vessel wall layers are intact but thinned and dilated. A pseudoaneurysm or a false aneurysm results from a tear in a vessel wall with a subsequent extravascular formation of hematoma adjacent to an injured arterial wall. A false wall forms from products of the clotting cascade, leading to localized turbulent flow and compression of nearby structures. The most common types of pseudoaneurysms are femoral, aortic, and cardiac. In rare cases, pseudoaneurysms can form in visceral organs or peripheral arteries [1]. The etiology of this disease can be iatrogenic following catheterization, biopsy, or surgery. By 2019, iatrogenic cases of pseudoaneurysm had an incidence of 0.44%-1.8% subsequent to diagnostic arterial catheterization and 3.2%-7.7% following intervention [2]. The cause can also be non-iatrogenic in the setting of trauma, infection, and vasculitides [3-5]. Early detection and treatment are critical due to the risk of rupture and consequent hemorrhagic shock, compartment syndrome, or muscle necrosis [1,6].

True and false gluteal artery aneurysms constitute less than 1% of all aneurysms, making pseudoaneurysms of the superior gluteal artery (SGA) an even rarer phenomenon [7]. Sporadic cases have been reported in the setting of penetrating trauma, pelvic fractures, orthopedic surgeries of the hip and femur, and perianal abscess drainage. Clinically, these patients typically present with an acutely painful and pulsatile mass, leading to rapid diagnosis and treatment [3,6,8,9]. However, rare subclinical symptoms may lead to misdiagnosis and dangerous complications.

This case report illustrates a rare presentation of pseudoaneurysm of the SGA following a minor traumatic fall at ground level, which was misdiagnosed due to its subclinical presentation. The patient's worsening symptoms after six months from the initial trauma rendered surgical intervention; however, an attempt to evacuate the presumed hematoma led to intraoperative hemorrhage and the discovery of a pseudoaneurysm. We aim to underscore the importance of awareness and increased clinical suspicion of pseudoaneurysm formation in minor trauma to ensure correct diagnosis and prevent life-threatening complications. The lack of literature on the formation of pseudoaneurysms of the SGA after a minor fall further emphasizes the importance of reporting this case.

## Case presentation

**Figure 1:**
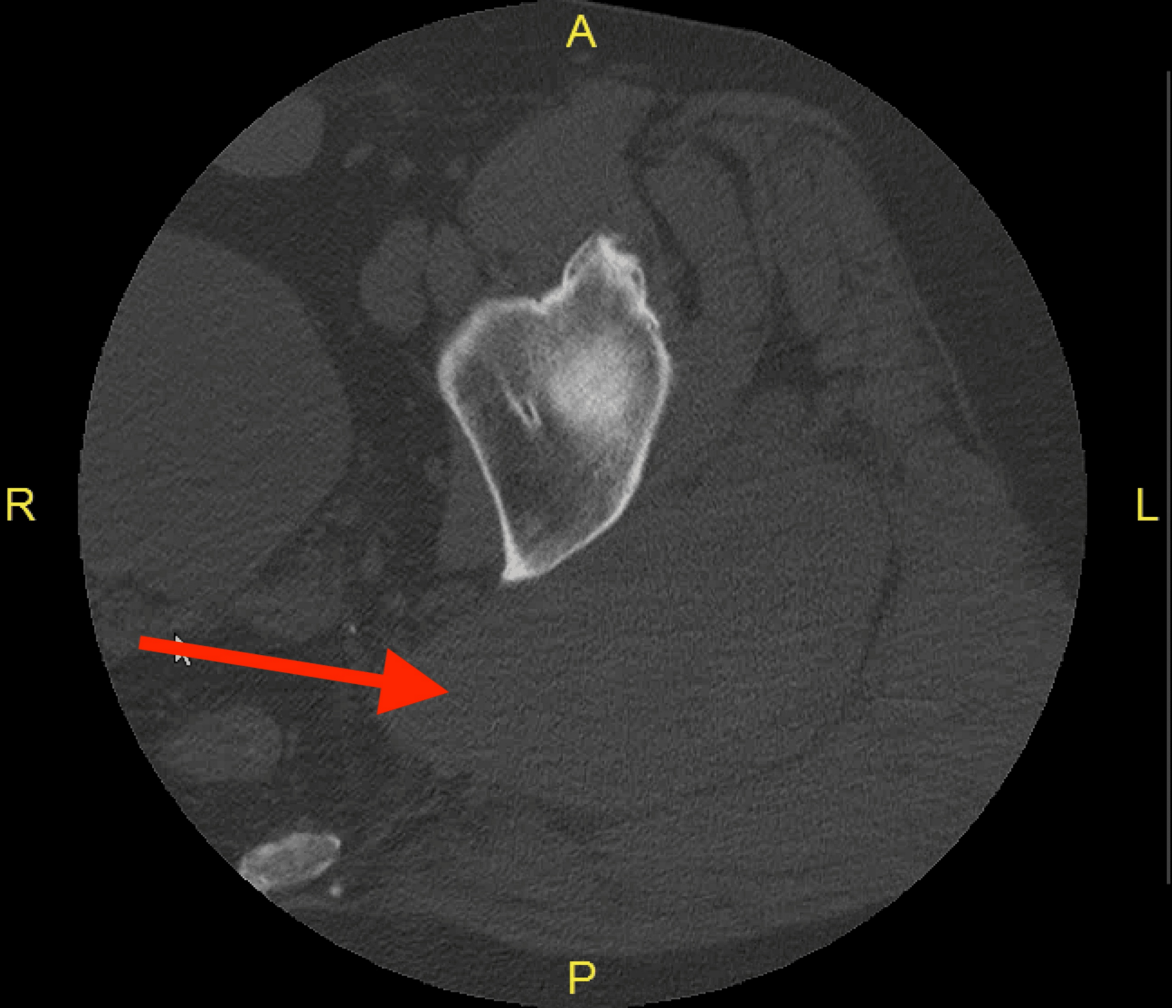
CT of the left lower extremity (axial view) There is no evidence of an acute fracture. Diffusely edematous left piriformis with a large intramuscular hematoma (red arrow) measuring 7.4 x 4 cm.

A 51-year-old male with a medical history of aortic insufficiency, hypertension, hyperlipidemia, and a surgical history of two aortic valve replacements (mechanical, now bovine) on aspirin 81 mg daily, amlodipine 10 mg daily, metoprolol 25 mg bidaily, and atorvastatin 40 mg daily presented to the emergency department (ED) complaining of left gluteal pain. The patient reported a history of a ground-level fall six months ago; he slipped on ice and fell on his backside. At that time, non-contrast CT of the left lower extremity (Figure 1) showed a large intramuscular hematoma measuring 7.4 x 4 cm, and a repeated same-day CT with contrast showed no size expansion. The patient was admitted for 24 hours of observation and managed conservatively without intervention for hematoma of the left gluteus. The patient stated that after the initial event, the bulge never disappeared, but the pain went away after two weeks, so he did not follow up further.

**Figure 2:**
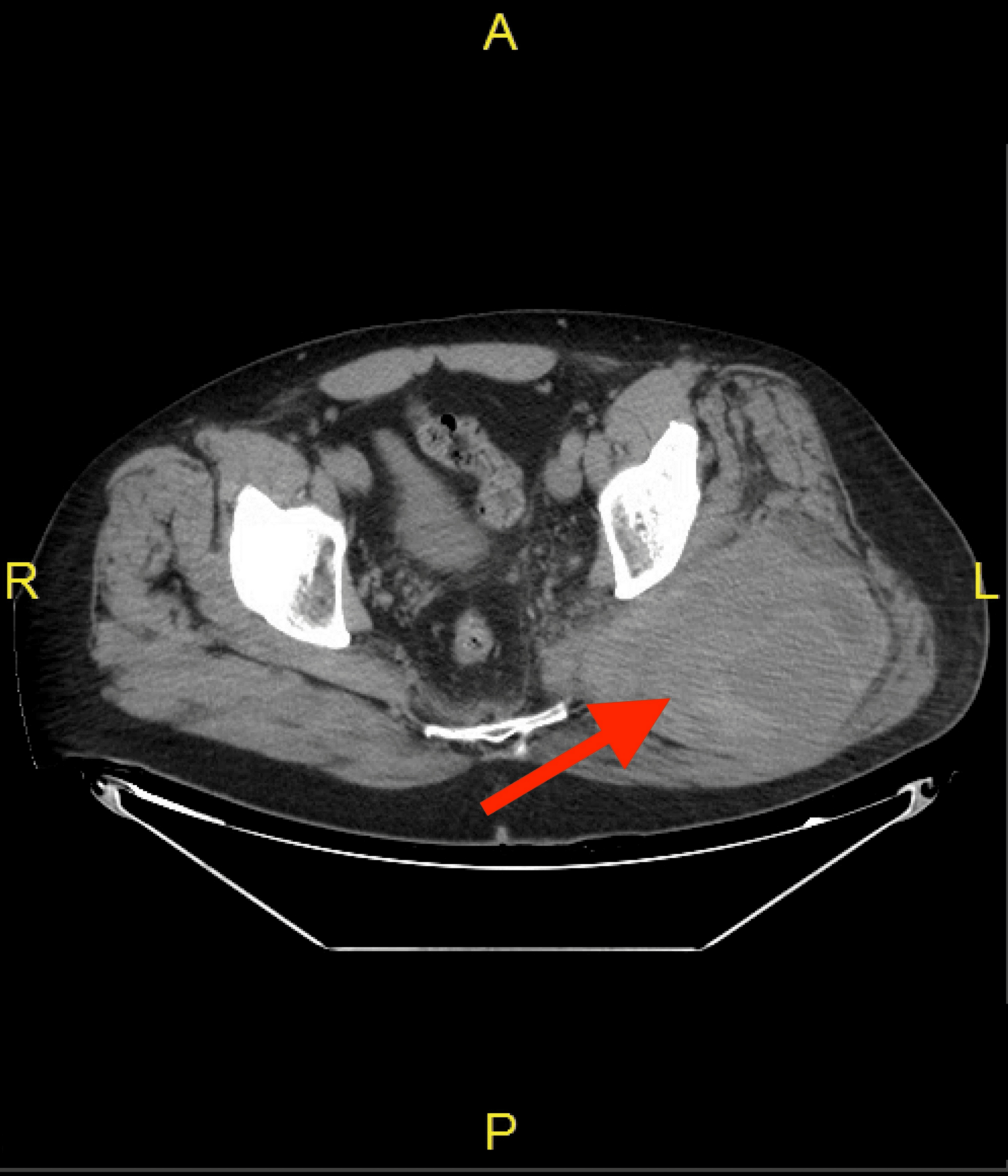
CT of the pelvis (axial view) No concerning bony lesions were identified. The left gluteal soft tissue has an enlarging, heterogeneous fluid collection (red arrow), approximately 10.8 x 14.6 x 15 cm, representing an expanding hematoma with blood pooling on venous phase imaging concerning active extravasation.

On the morning of the current presentation, the pain started when the patient was gardening and worsened when sitting directly on the bulge or walking. A physical exam revealed a large area of firmness and swelling on the left gluteus compared to the right, with no skin changes or pulsations. The lower extremity compartments were soft bilaterally with strong, palpable peripheral pulses and intact motor and sensory functions. The patient was afebrile and hemodynamically stable; laboratory results (Table 1) showed hemoglobin (Hgb) levels of 12.9 g/dL, an international normalized ratio (INR) of 1.25, prothrombin time (PT) of 15.6 seconds, and partial thromboplastin time (PTT) of 34.5 seconds. The CT of the pelvis with contrast (Figure 2) revealed an expanding left gluteal hematoma measuring 10.8 x 14.6 x 15 cm with pooling of contrast on delayed phase imaging concerning active extravasation. A repeated same-day CT showed a stable hematoma of unchanged size, and the patient was admitted to the hospital for close clinical observations. The patient was observed for four days on the surgical floor, during which his vitals, labs, and physical exam remained stable and did not suggest any active bleeding, which led us to believe it was a stable hematoma of the left gluteus. The size of the presumed hematoma remained unchanged, but the pain persisted. Due to its highly symptomatic nature, the decision was made to take the patient into the operating room (OR) for hematoma evacuation with a preoperative diagnosis of left buttock hematoma. Risks, benefits, and alternatives were discussed with the patient, and informed consent was obtained.

**Table 1:** Laboratory values procured on the initial presentation in the emergency department WBC: white blood cells; PT: prothrombin time; INR: international normalized ratio; APTT: activated partial thromboplastin time

Investigation	Patient’s Laboratory Values	Reference Ranges
WBC	8.7 K/uL	4.0-11.2 K/uL
Hemoglobin	12.9 g/dL	13.7-17.5 g/dL
Hematocrit	39.7%	40.0-51.0%
Platelet count	224 k/uL	150-400 k/uL
PT	15.6 seconds	12-14.8 seconds
INR	1.25	0.9-1.2
APTT	34.5 seconds	22.8-36.5 seconds

In the OR, a longitudinal incision was made over the posterolateral portion of the left buttock. It was deepened down to the gluteus muscles, which were split parallel to the fibers, exposing the hematoma capsule. An 18-gauge needle was inserted into the capsule, and an old clot was aspirated. Then, using a 15-blade scalpel, the capsule was incised. Upon doing so, there was an explosive return of bright red arterial blood. The wound was immediately packed, and pressure was held. The bleeding persisted upon packing removal, and we were unable to identify a specific vessel. The edges of the capsule were approximated with several figure-of-eight stitches, which allowed for temporary control of the bleeding. The wound was packed with gauze, and a sterile dressing was applied.

**Figure 3:**
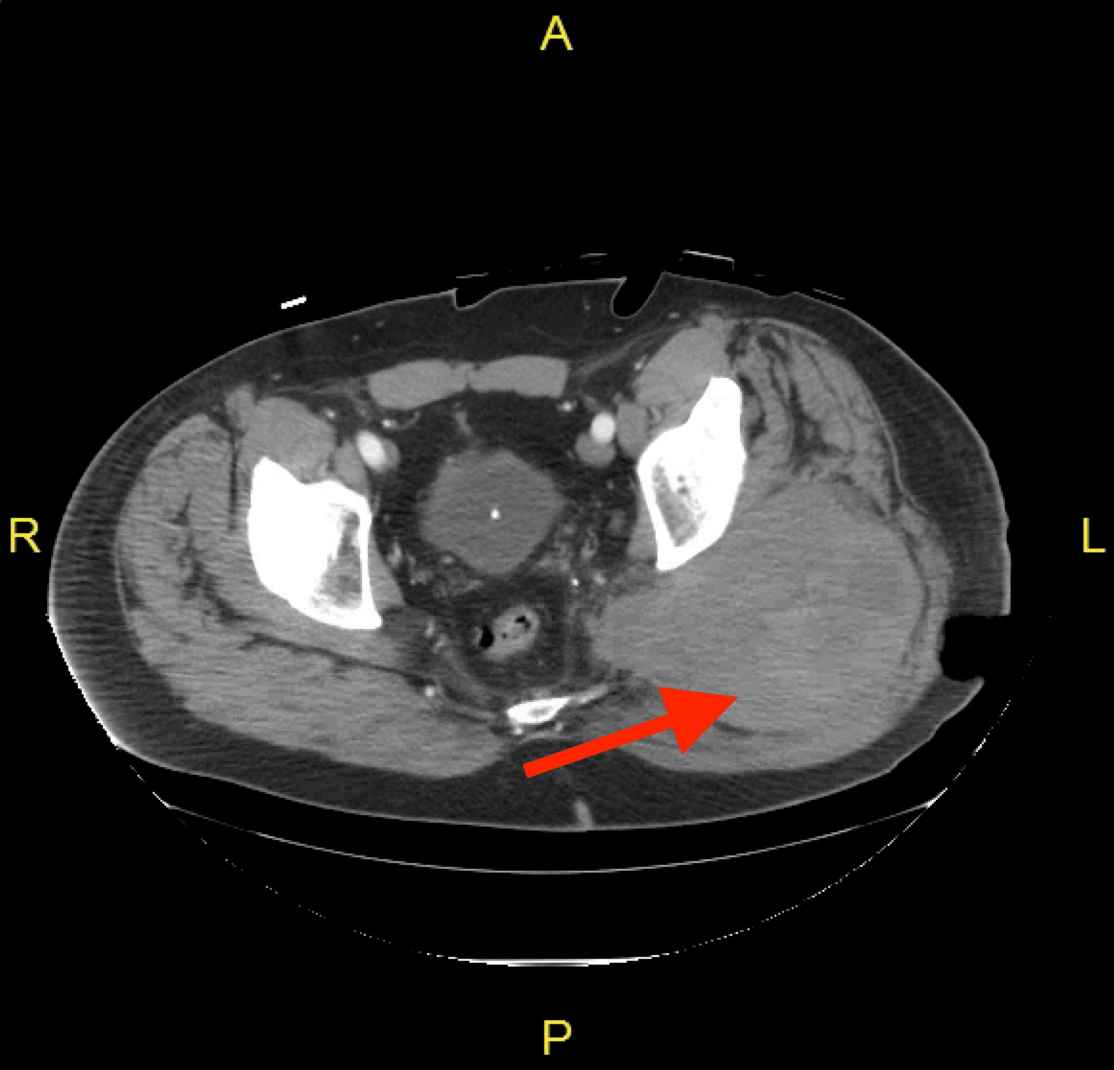
Abdominal/pelvis CT angiography (axial view) No acute fractures. A large, well-circumscribed hyperdense mass (red arrow), measuring 12.7 x 14.3 cm, is seen posteriorly to the left hip. The impression is of a large hematoma posterior to the left hip joint, with active enhancement consistent with active hemorrhage.

**Figure 4:**
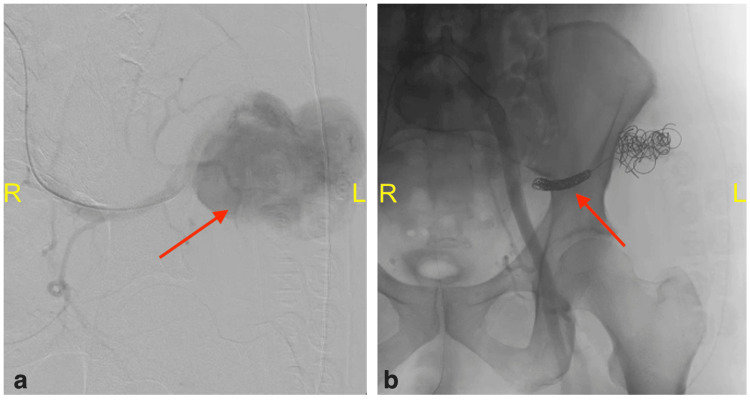
Pelvic angiogram of the left superior gluteal artery (coronal view) (a) Pre-embolization angiogram of the left superior gluteal artery with visualization of a pseudoaneurysm (red arrow); (b) post-coil embolization angiogram with discontinuation of outflow from the left superior gluteal artery to pseudoaneurysm; no extravasation and no retrograde filling after coil placement (red arrow).

The intubated patient was then taken for a CT angiography of the abdomen and pelvis, which showed active extravasation of blood (Figure 3). Subsequently, the patient was taken to the interventional radiology (IR) suite, where an angiogram of the SGA confirmed a large pseudoaneurysm (Figure 4a), which was subsequently embolized (Figure 4b) successfully to control the bleeding. The total estimated blood loss during the procedure was 3 L. The patient was admitted postoperatively to the surgical intensive care unit (SICU) in a guarded condition secondary to acute blood loss anemia. On postoperative day (POD) one, the patient was successfully extubated. On POD3, the patient was stable with no signs of active arterial bleeding and was discharged from SICU to a regular floor. On POD5, the patient was taken back to the OR for indicated evacuation of left buttock hematoma, which was not possible during the index case due to previously mentioned complications. Approximately 300 mL of old clots were removed along with the hematoma capsule. With the clot evacuated, the wound was packed with gauze, and a sterile dressing was applied. The patient did well postoperatively. The wound was managed with dressing changes and wound vac placement. On POD7, the patient was discharged home with appropriate home wound care services, strict return precautions, and a follow-up appointment at the outpatient surgery clinic.

## Discussion

Gluteal pseudoaneurysms account for less than 1% of all aneurysms [4,9]. They most commonly occur following severe trauma, with blunt and penetrating pelvic trauma being the leading causes [3-5]. In very rare events, such as ours, pseudoaneurysms may develop following minor trauma or no trauma at all. Our patient developed an SGA pseudoaneurysm after a minor traumatic fall at ground level and presented with subclinical symptoms for six months. One working theory for this uncommon presentation could be the patient's history of antiplatelet therapy. A case report from 2008 documented a similar inferior gluteal artery injury following a minor fall in a patient who was also on antiplatelet medication at the time of injury [10]. Another case report from 2005 documented a 78-year-old female who presented with an inferior gluteal artery pseudoaneurysm with no history of trauma but a positive history of antiplatelet therapy [11]. Therefore, it is possible that patients who are on antiplatelet therapy should have a higher level of suspicion for pseudoaneurysm formation following minor trauma.

Another notable point in this rare case is the atypical clinical presentation of SGA pseudoaneurysm. Pseudoaneurysm commonly presents as a painful mass accompanied by pulsation and a bruit on auscultation [4,12]. However, our patient presented with a stable swelling in the gluteal region in the absence of a bruit and pulsation on both ED visits. The absence of a bruit or pulsation increases the chances of misdiagnosing pseudoaneurysm and may be absent when a small pseudoaneurysm is lined with a thrombus or encased by a hematoma [3].

Moreover, our patient experienced transient pain in the left gluteal region right after the traumatic fall, which resolved after two weeks but then reemerged six months later. As the size of the pseudoaneurysm increases, surrounding structures, such as the nerves, including the sciatic nerve, result in limb pain and paresthesia [2,8]. Neurological symptoms can extend to urinary and bowel dysfunction due to the course of the SGA between the lumbosacral trunk and the first sacral ventral ramus [8]. Pseudoaneurysm can also compress adjacent veins and arteries and the surrounding skin. Compression of these structures typically results in leg swelling, pain, rubor, and, in later stages, loss of distal pulse, necrosis, and limb and skin ischemia [1,2]. In the span of six months, our patient's pseudoaneurysm increased from 7.4 × 4 cm to 10.8 x 14.6 × 15 cm. However, despite this expansion, the patient exhibited none of the previously mentioned compression symptoms, presenting clinically with soft lower extremity compartments, intact pulses, and intact motor and sensory functions.

Treatment for our patient was complicated by the initial misdiagnosis of the SGA pseudoaneurysm as a hematoma of the left gluteus. Initially, an attempt was made to evacuate the presumed hematoma, which led to uncontrolled arterial bleeding and the discovery of SGA pseudoaneurysm. This major complication resulted in 3 L of blood loss and required emergent embolization of SGA. In typical cases of pseudoaneurysm, two types of procedures can be performed: open surgery and endovascular therapy, including embolization, ultrasound-guided thrombin injection, and surgery with arterial ligation [3,8]. Endovascular procedures for treating pseudoaneurysm decrease the complications of open surgical treatment and have been the first-line treatment for patients with uncomplicated pseudoaneurysm. This enables a faster recovery time and a decrease in the length of hospital stay [3,13].

In many cases, endovascular embolization is the preferred approach because it involves using coils to occlude the pseudoaneurysm and control bleeding utilizing a minimally invasive method. Surgical intervention, including direct repair or arterial ligation, is performed when endovascular methods are not accessible or show no efficacy and the symptoms and pseudoaneurysm persist. Ultrasound-guided thrombin injection is used for smaller pseudoaneurysms (less than 3-4 cm in diameter) but is not commonly used for SGA lesions [14]. Conservative management with close monitoring is reserved for stable, asymptomatic cases.

## Conclusions

The occurrence of a pseudoaneurysm in the SGA following a minor traumatic event is exceedingly rare. This case stands out not only because of its unusual location but also because the hallmark clinical signs typically associated with pseudoaneurysms are completely absent, making diagnosis even more challenging. We present this case to underscore the need for heightened clinical suspicion of pseudoaneurysm, even in the context of seemingly minor trauma. Our aim is to equip clinicians with greater awareness of this potential complication, thereby reducing the likelihood of misdiagnosis.
